# An antibody‐drug conjugate targeting a GSTA glycosite‐signature epitope of MUC1 expressed by non‐small cell lung cancer

**DOI:** 10.1002/cam4.3554

**Published:** 2020-10-20

**Authors:** Deng Pan, Yubo Tang, Jiao Tong, Chengmei Xie, Jiaxi Chen, Chunchao Feng, Patrick Hwu, Wei Huang, Dapeng Zhou

**Affiliations:** ^1^ Tongji University School of Medicine Shanghai China; ^2^ Shanghai Pudong New Area Mental Health Center affiliated with Tongji University School of Medicine Shanghai China; ^3^ CAS Key Laboratory of Receptor Research CAS Center for Excellence in Molecular Cell Science Shanghai Institute of Materia Medica Chinese Academy of Sciences Shanghai China; ^4^ Department of Melanoma Medical Oncology The University of Texas MD Anderson Cancer Center Houston TX USA

**Keywords:** aberrant glycosylation, antibody‐drug conjugate, glycosite, MUC1, neoantigen

## Abstract

Antibodies targeting aberrantly glycosylated proteins are ineffective in treating cancer. Antibody‐drug conjugates have emerged as effective alternatives, facilitating tumor‐specific drug delivery. Previous studies have assessed the aberrantly glycosylated tandem repeat region of MUC1 glycoprotein as three site‐specific glycosylated neoantigen peptide motifs (PDTR, GSTA, and GVTS) for binding with a monoclonal antibody. This study aimed to develop an antibody‐drug conjugate for cancer treatment based on monoclonal antibodies against the aforementioned three neoantigen peptide motifs. Internalization of monoclonal antibodies was assessed via immunofluorescence staining and colocalization with lysosomal markers in live cells. Antibody positivity in tumor and peritumoral tissue samples was assessed via immunohistochemistry. The efficacy of anti‐MUC1 ADCs was evaluated using various cancer cell lines and a mouse tumor xenograft model. An anti‐MUC1 ADC was synthesized by conjugating GSTA neoantigen‐specific 16A with monomethyl auristatin E (MMAE), which displayed potent antitumoral efficacy with an IC_50_ ranging 0.2–49.4 nM toward various cancer cells. In vivo, 16A‐MMAE inhibited tumor growth in a dose‐dependent manner in a mouse xenograft model established using the NCI‐H838 NSCLC cell line, at a minimum effective dose of 1 mg/kg. At 3 mg/kg, 16A‐MMAE did not cause significant toxicity in a transgenic mouse expressing human MUC1. The high antitumoral efficacy of 16A‐MMAE suggests that aberrant glycosylated MUC1 neoantigen is a potential target for the development of ADCs for treating various cancers. Personalized therapy may be achieved through such glycosite‐specific ADCs.

## Graphical abstract

In this study, we describe an anti‐MUC1 antibody‐drug conjugate, 16A‐monomethyl auristatin E (MMAE). 16A‐MMAE showed potent antitumoral activity in multiple types of cancer, suggesting that neoantigens generated by posttranslational modification are promising targets for antibody‐drug development.

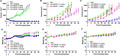

## 1 INTRODUCTION

Mucin‐1 (MUC1), also known as EMA, PEM, or CA15‐3 antigen, is a transmembrane glycoprotein that is considered a prominent target for tumor immunotherapy.^1^, ^2^ In healthy cells, MUC1 is extensively O‐glycosylated. The extracellular portion contains a variable number of tandem repeats (VNTR), which typically range 20–120. Each TR consists of 20 amino acid residues along with five potential O‐glycosylation sites.^3^, ^4^ MUC1 is expressed in almost all epithelial cancers.^5^, ^6^, ^7^, ^8^ MUC1 differs between tumor and normal cells, the former characterized by aberrant, truncated glycosylation, yielding glycopeptide epitopes that can be recognized by specific antibodies. Since such glycopeptide epitopes are tumor‐specific, they may represent potential targets for therapeutic antibodies. However, monoclonal antibody therapeutics targeting MUC1 have not been proven effective in clinical trials.^9^, ^10^ It has been hypothesized that the MUC1 subunit harboring the TRs circulates at high levels among cancer patients and serves as a “sink” precluding the delivery of antibodies to the tumor cell surface. However, significant inhibition of circulating MUC1 on antibody‐dependent cellular cytotoxicity has been reported exclusively in the serum of patients with MUC1 levels of >100 U/ml.^11^ High levels of circulating autoantibodies against both the cancer‐specific MUC1 isoform and the non‐glycosylated signal peptide domain of MUC1 (up to 200 μg/ml) have been reported in human cancers.^12^ Autoantibodies against a single glycopeptide epitope in cancer patients is highly variable.^13^ Furthermore, autoantibodies against aberrantly glycosylated MUC1 in early‐stage breast cancer are associated with a better prognosis.^14^ However, it remains unclear whether autoantibodies against MUC1 affect the efficacy and specific targeting of antibody drugs. No studies have assessed the affinity of autoantibodies toward MUC1. Accordingly, high affinity is an essential criterion for the development of therapeutic antibodies.

Recent efforts have evaluated the potential of MUC1 as a candidate for the development of antibody‐drug conjugates (ADCs). An ADC consists of three components: an antibody, an antitumoral agent, and a linker. The first ADC approved by the FDA was gemtuzumab ozogamicin (Mylotarg), a humanized anti‐CD33 IgG4 antibody conjugated with calicheamicin, a potent cytotoxic agent causing double‐strand DNA breaks.^15^ It was used to treat patients with relapsed acute myeloid leukemia. Currently, two other FDA‐approved ADCs are clinically available. Brentuximab vedotin (Adcetris) is an anti‐CD30 antibody linked to an MMAE, an antimitotic agent inhibiting cell division by blocking tubulin polymerization. It has been approved for treating relapsed or refractory systemic anaplastic large‐cell lymphoma or Hodgkin's lymphoma.^16^ Trastuzumab emtansine (Kadcyla) is an anti‐Her2 antibody linked to the tubulin inhibitor maytansine derivative DM1 (T‐DM1). It is used for treating advanced Her2‐positive breast cancer.^17^ Furthermore, >30 ADCs targeting various blood tumors and solid carcinomas are being clinically developed.^18^

We and others have assessed the aberrantly glycosylated TR region as neoantigen peptide motifs (PDTR, GSTA, and GVTS) for monoclonal antibody binding.^2^, ^19^ In this study, we screened monoclonal antibodies specific to the aforementioned three neoantigen peptide motifs and synthesized antibody‐MMAE for cancer treatment.

## 2 MATERIALS AND METHODS

### 2.1 Cell lines and reagents

Human tumor cell lines NCI‐H838 (H838), NCI‐H2030 (H2030), NCI‐H1650 (H1650), NCI‐H1975 (H1975), NCI‐H23 (H23), NCI‐H520 (H520), NCI‐H460 (H460), NCI‐H292 (H292), NCI‐H1229 (H1229), A549, PC9, MCF‐7, SKBR3, PANC‐1, CFPAC1, N87, HGC‐27, H8910, SKOV3, ES2, Hey, and KGN (obtained from the America Type Culture Collection, ATCC) were cultured at 37°C with 5% of CO_2_ in RPMI‐1640 or DMEM media (Life Technologies) supplemented with 10% of fetal bovine serum (FBS, Life Technologies). Endo‐S was expressed in *E*.*coli* through a previously described protocol.^20^ MMAE and Fmoc‐Val‐Cit (valine‐citrulline)‐PAB‐PNP were purchased from Levena Biopharma (Nanjing, China). Other chemical reagents and solvents were purchased from Sinopharm Chemical Reagent Co. (Shanghai, China) or Sigma‐Aldrich and used without further purification. The MAbPac RP column (4 μm, 3.0 × 100 mm^2^) was purchased from Thermo Fisher Scientific. Nuclear magnetic resonance (NMR) spectra were assessed using a Varian‐MERCURY Plus‐500 instrument (Agilent). High‐resolution electrospray ionization mass spectra were measured using an 6230 LC‐TOF MS spectrometer (Agilent).

### 2.2 Confocal microscopy

H838 cells were seeded on glass cover slips in 3.5‐cm dishes (1.5 × 10^5^ cells per well) and cultured at 37°C with 5% of CO_2_ in RPMI‐1640 supplemented with 10% of fetal bovine serum for 24 h. Cells were incubated with 2 μg/ml cy5‐labeled 16A monoclonal antibody^21^ (mAb) for 3.5 h and subsequently incubated with a 75 nM lysosome fluorescent probe (LysoTracker Red DND 99, Thermo Fisher Scientific) for 30 min. Thereafter, cells were washed, fixed, and observed using a confocal laser scanning microscope (Nikon A1R).

### 2.3 Internalization of the 16A antibody

To assess antibody internalization, multiple sets of 2 × 10^5^ H838 cells were first incubated with unlabeled 16A mAb at a saturating concentration of 5 μg/ml. The negative control group without internalization was allowed to stand on ice for 150 min. For internalization experiments, other sets of antibody‐coated cells were incubated at 37°C to facilitate antibody internalization for different periods (30, 60, 90, 120, and 150 min). Thereafter, cells were washed with ice‐cold PBS buffer and stained with a PE‐labeled anti‐mouse IgG secondary antibody (Southern Biotech) at a 1 μg/ml concentration for 30 min on ice. After three washes with PBS, the cells were harvested and subjected to flow cytometry analysis to determine the mean fluorescence intensity (MFI). The amount of 16A antibody internalized into cells at each time point was determined from the percentage reduction in the MFI relative to control cells incubated at 4°C for 150 min.

### 2.4 Flow cytometry staining of cancer cell lines

Cell surface expression of MUC1 was assessed through flow cytometry staining. The cells were washed with 2% of BSA in PBS, and then, incubated with the 16A,^21^ SM3, or C595 antibody (Abcam) at 5 µg/ml for 30 min at 4°C. After washing, cells were incubated with PE‐labeled anti‐mouse IgG (1 µg/ml) for 30 min at 4°C. After washing, cells were analyzed using FACS Calibur (BD), and the data were analyzed using FlowJo software (version 7.6, BD).

### 2.5 Immunohistochemistry

Tumor tissue array slides were obtained from CrownBio. Immunohistochemistry was performed using Bond RX automatic IHC&ISH machine, Leica. In brief, paraffin‐embedded tissue samples were treated with Dewax solution and antigen retrieval buffer sequentially, and then, probed with primary and secondary antibodies. Stained sections were scanned using the NanoZoomer Image system. IHC staining intensity was scored at four levels: 0 (negative), 1 (weak staining), 2 (medium staining), and 3 (strong staining). Tumor cell percentages at different intensity levels were then evaluated.

TotalScore=(%at0)×0+(%at1)×1+(%at2)×2+(%at3)×3.

### 2.6 Synthesis of NHS‐Val‐Cit‐PAB‐MMAE

NH_2_‐Val‐Cit‐PAB‐MMAE (29.7 mM in 300 μl DMF) was added gradually (30 μl every 15 min) to a solution of disuccinimidyl glutarate (DSG, 53.4 mM) prepared in a mixture (1:1 volume) of DMF/phosphate buffer (50 mM, pH 7.5). The reaction mixture was stirred at 25°C for 3 h and monitored through RP‐HPLC. The product was purified through preparative HPLC to yield a white powder (9.1 mg, 76.5%). 1H‐NMR (500 MHz, DMSO‐d6) δ 8.14 (d, J = 7.5 Hz, 1H), 7.89 (d, J = 8.6 Hz, 2H), 7.59 (d, J = 8.1 Hz, 2H), 7.32 (d, J = 7.8 Hz, 2H), 7.28 (dd, J = 7.5, 2.7 Hz, 2H), 7.17 (s, 1H), 5.98 (s, 1H), 5.04 (dd, J = 31.4, 17.1 Hz, 3H), 4.50 (d, J = 5.9 Hz, 1H), 4.44 (d, J = 6.6 Hz, 1H), 4.39 (d, J = 8.3 Hz, 1H), 4.31–4.26 (m, 1H), 4.26–4.18 (m, 2H), 4.06–3.93 (m, 3H), 3.61–3.55 (m, 2H), 3.25 (d, J = 7.7 Hz, 3H), 3.20 (d, J = 12.5 Hz, 3H), 3.12 (s, 1H), 2.98 (s, 1H), 2.87 (dd, J = 18.5, 5.7 Hz, 3H), 2.82 (s, 3H), 2.69 (d, J = 7.5 Hz, 2H), 2.42 (d, J = 16.2 Hz, 2H), 2.36–2.23 (m, 4H), 2.17–2.08 (m, 2H), 2.01–1.68 (m, 10H), 1.63–1.47 (m, 4H), 1.07–0.98 (m, 6H), 0.91–0.74 (m, 21H). 13C NMR (126 MHz, DMSO‐d6) δ 171.42, 171.16, 170.54, 170.20, 168.71, 158.82, 143.61, 128.11, 127.74, 127.68, 126.68, 126.60, 126.40, 126.35, 85.37, 74.74, 60.87, 58.12, 57.55, 57.10, 54.93, 54.06, 53.10, 47.15, 46.19, 43.70, 43.15, 38.55, 33.58, 31.48, 30.37, 29.87, 29.63, 29.25, 26.78, 25.41, 25.30, 24.29, 23.07, 20.56, 19.19, 18.88, 18.12, 15.41, 15.24, 14.96, 10.26. The calculated HRMS for [M + H]+was 1334.7612, and the observed HRMS was 1334.7586; [M+Na]+, calculated HRMS, 1356.7931; observed HRMS, 1356.7399.

### 2.7 Preparation of lysine‐linked ADCs

16A monoclonal antibody (1 mg/ml) and NHS‐Val‐Cit‐PAB‐MMAE (1.5 mM) in phosphate buffer (pH 8.0, 50 mM) containing 4%–5% of DMSO was incubated at 37°C for 2 h. The reaction mixture was immediately placed in a protein A affinity chromatography column for purification. Before loading the ADC sample, the protein A‐agarose column was prewashed with glycine‐HCl (100 mM, pH 2.5, 5 column volumes) and pre‐equilibrated with phosphate buffer (50 mM, pH 8.0, 5 column volumes). After loading the ADC reaction mixture, the column was washed with phosphate buffer (50 mM, pH 8.0, 5 column volumes) and glycine‐HCl (20 mM, pH 5.0, 3 column volumes) successively. Thereafter, the bound ADC was eluted with glycine‐HCl (100 mM, pH 2.5, 5 column volumes), followed immediately by neutralization to pH 7.5 with glycine‐HCl (1 M, pH 8.8). The fractions containing the target ADC were combined and concentrated through centrifugal filtration through a 10‐kDa cutoff membrane.

### 2.8 Liquid chromatography–mass spectrometry (LC‐MS)

Small‐molecule ESI‐MS spectra were measured using an 6230 LC‐TOF MS spectrometer (Agilent). The small molecules were analyzed using a short‐guard column and eluted with 70% of methanol containing 0.1% of formic acid. The mass spectra of small molecules were recorded in the mass range of 200–3000 or 600–2000 under the HRMS mode (standard 3200 m/z, 4 GHz). Key source parameters: drying nitrogen gas flow, 11 l/min; nebulizer pressure, 40 psi; gas temperature, 350°C; fragment or voltage, 175 V; skimmer voltage, 65 V; and capillary voltage, 4000 V.

LC‐MS spectra of the antibodies and ADCs were measured using the same MS spectrometer (Agilent 6230), using a THERMO MAbPac RP column (4 μm, 3.0 × 100 mm^2^) at 80°C. Elution was carried out using an isocratic mobile phase of 20% acetonitrile (Buffer B) and 80% water containing 0.1% of formic acid (Buffer A) for the first 3 min at a flow rate of 0.4 ml/min; thereafter, it was successively eluted at the same flow rate along a linear gradient of 20%–50% acetonitrile for additional 2.5 min, an isocratic 50% acetonitrile solution for 2 min, another linear gradient of 50%–90% acetonitrile for 0.5 min, and an isocratic 90% acetonitrile solution for 2 min. The mass spectra of the antibodies were obtained under an extended mass range mode (high 20,000 m/z, 1 GHz) in the mass range of 800–5000. Key source parameters: a drying nitrogen gas flow of 11 L/min; a nebulizer pressure of 60 psi; a gas temperature of 350°C; a fragment or voltage of 400 V; a skimmer voltage of 65 V; and a capillary voltage of 5000 V. Multiple charged peaks of the antibody were deconvoluted using the Agilent MassHunter BioConfirm software (deconvolution for protein, Agilent technology) at a deconvolution range of 20–160 kDa; other parameters were set at default values for protein deconvolution. The TOF was calibrated over a range of 0–6000 m/z using Agilent ESI calibration mix solution before analysis. The peak of MS 922 is the internal standard for calibration.

### 2.9 Pharmacokinetic studies of ADC

All animal studies were approved by the animal care and use committee of Tongji University, Shanghai, China. All experiments were carried out in SPF‐controlled housing facilities. Groups of mice (*n* = 3, C57BL/6 strain, 8 weeks old) were injected intravenously with a single dose of nonconjugated 16A mAb or 16A‐MMAE at 5 mg/kg (mean body weight of mice being 20 g). Whole blood was sampled from the tail vein at various time points (0.25, 6, 24, 48, 96, 144, and 192 h after treatment). Serum was obtained through centrifugation and stored at −80°C until analysis.

The antibody‐drug concentrations were assessed via ELISA, in 96‐well plates coated with 1.5 μg/ml of streptavidin (S4762) at 4°C overnight. The plate was blocked with 1% of bovine serum albumin in PBS at 37°C for 1 h and incubated with 2 μg/ml of biotinylated MUC1 glycopeptide antigen at 37°C for 1 h. After washing, the mouse plasma samples and calibration standards of 16A/16A‐MMAE (serial concentration: 400 to 3.125 ng/ml) were added. The binding reaction of the plasma antibody drug was carried out for 1 h at 37°C. After washing, the plate was incubated with horseradish peroxidase (HRP)‐conjugated anti‐mouse IgG at 37°C for 1 h and visualized with 100 µl of TMB substrate for 30 min at room temperature, and the reaction was terminated with 100 µl of the termination solution. The absorbance was measured at 450 nm. Antibody‐drug concentrations and pharmacokinetic parameters were determined using the PK solver software.^22^

### 2.10 Cytotoxicity assay

The 3‐(4,5‐dimethyl‐2‐thiazolyl)‐2,5‐diphenyl‐2‐H‐tetrazolium bromide (MTT) assay was performed to determine the in vitro efficacy of the ADCs. Briefly, cells were plated at 5000 cells per well in 96‐well plates at 37°C and 5% CO_2_ overnight. The ADC samples were serially diluted from 100 to 10^−4 ^μg/ml in culture medium. Cell viability was assessed after 72 h via the MTT assay. The IC_50_ was determined using GraphPad Prism version 5 (GraphPad Software, Inc.).

### 2.11 Efficacy of 16A‐MMAE in mouse xenograft tumor models

Groups of BALB/c nu/nu mice (6‐week‐old, female) were inoculated with 1 × 10^7^ H838 cells in 200 μl of PBS on the right flank. Tumor size was measured from day 8 (day 7 after initial injection), and then, every 2–3 days. The longest length (*a*) and the length perpendicular to the longest length (*b*) were considered in the formula *V* = ½ × *a *× (*b*)^2^ to determine the tumor volume in mm^3^. Mice were randomized into different treatment groups (*n* = 5) when tumors approached 150–250 mm^3^ and treated with antibody drugs.

The different treatment groups included 16A (15 mg/kg), 16A‐MMAE (0.5, 1, 3, 5, and 15 mg/kg), and vehicle PBS, respectively. Mice received one or two doses of ADCs (the second dose was administered 48 h after the first dose), nonconjugated antibodies, or vehicle PBS through intravenous injection. Statistical analysis was performed using GraphPad Prism version 5 (GraphPad Software, Inc.).

### 2.12 16A‐MMAE toxicity in hMUC1 transgenic mice

Groups of hMUC1‐Tg mice^23^ (024631‐C57BL/6‐Tg (MUC1) 79.24Gend/J, 8‐week‐old, *n* = 6 per group, three male and three female, Jackson Laboratory, Bar Harbor, ME) were treated with 16A‐MMAE via the tail vein at 0, 3, 15, or 30 mg/kg. Systemic tissue toxicity was examined at the Shanghai Institute of Materia Medica's Center for Drug Safety Evaluation and Research. Clinicopathological parameters were assessed on days 3, 14, and 28. Tissue samples of the heart, liver, spleen, lung, kidney, gastric, pancreatic, and small intestine from ADC‐treated hMUC1 transgenic mice were fixed in 4% of formaldehyde and embedded in paraffin blocks, and then, stained with hematoxylin and eosin.

### 2.13 Efficacy of 16A‐MMAE on MUC1‐expressing syngeneic tumors in hMUC1 transgenic mice

A B16‐OVA‐hMUC1 cell line was generated through stable transfection of the B16‐OVA cell line^24^ with a pcMV3‐hygro(R) plasmid (Sino Biological, Beijing, China) encoding human *MUC1*. Groups of hMUC1‐Tg mice^23^ (024631‐C57BL/6‐Tg(MUC1)79.24Gend/J, 8‐week‐old, from Jackson Laboratory) were inoculated with 1 × 10^5^ B16‐OVA‐hMUC1 cells in 200 μl of PBS on the right flank. Mice were randomized into different treatment groups (*n* = 4) when tumors approached approximately 100 mm^3^. The treatment groups included 16A‐MMAE (3 and 10 mg/kg) and the vehicle PBS. Mice received two doses of 16A‐MMAE (the second dose was administered 48 h after the first dose) or the vehicle PBS through intravenous injection.

## 3 RESULTS

### 3.1 Internalization and delivery of the 16A antibody to lysosomes

**Figure 1:**
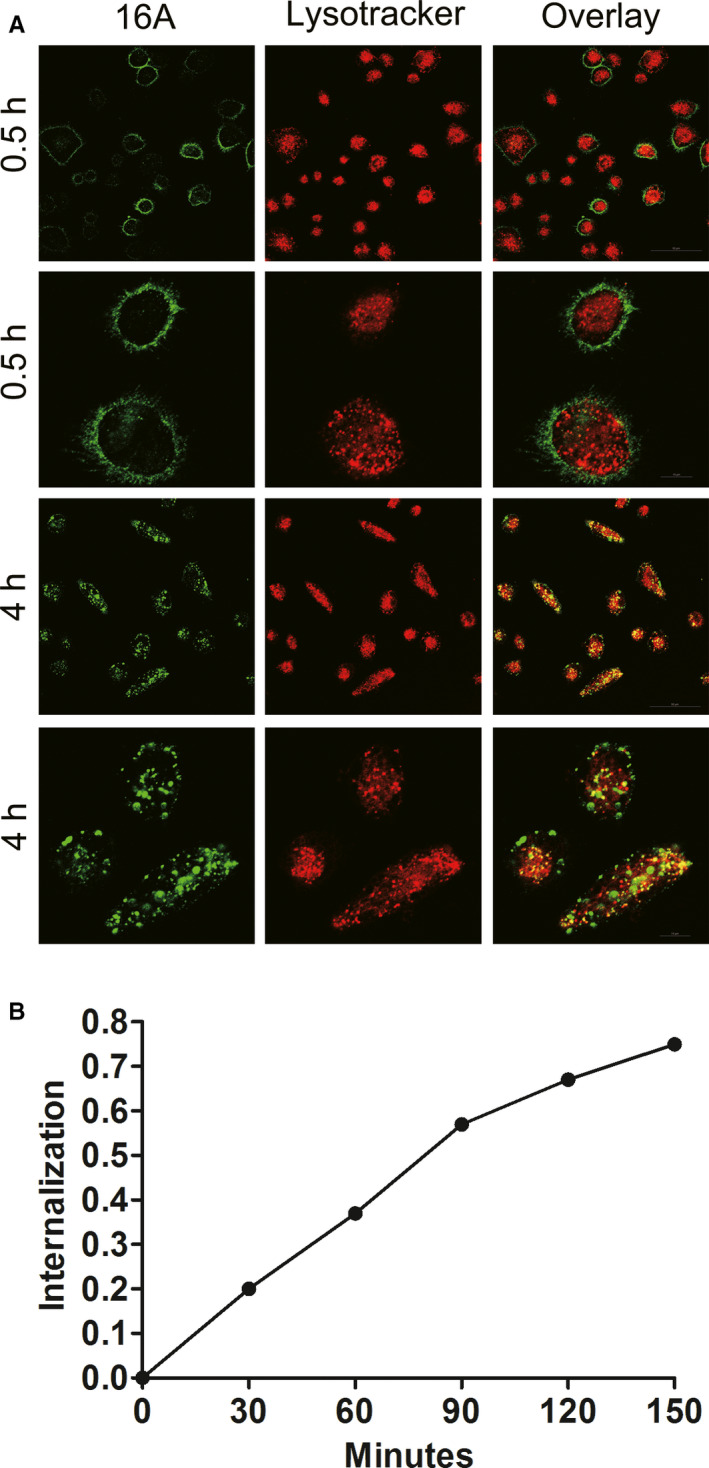
Internalization of the 16A antibody. (A) Fluorescence‐labeled 16A was incubated with H838 cells for 0.5 or 4 h at 37°C. After 0.5 h, the 16A antibody was localized to the cell membrane. Four hours later, the antibody was endocytosed and co‐localized with LysoTracker Red, a marker for acidic organelles in live cells. (B) Time course of 16A antibody internalization by lung cancer cell line H838 was assessed through flow cytometry.

We screened a panel of mAbs specific to aberrantly glycosylated MUC1 peptide motifs (PDTR, GSTA, and GVTS) and focused on 16A mAb.^21^ Cy5‐labeled 16A was initially localized on the cell surface after 30 min of incubation at 37°C (Figure 1A, green). After 4 h of incubation at 37°C, 16A was effectively internalized through endocytosis. Intracellular 16A co‐localized with LysoTracker Red DND 99, a red‐fluorescent dye for labeling and tracking acidic organelles in live cells, indicating that 16A was internalized and transported to the lysosomes (Figure 1A, yellow). Lung cancer cell line H838 internalized 16A antibody within 150 min (Figure 1B).

### 3.2 Positivity of the 16A antibody epitope in cancer cells and tissues

**Figure 2:**
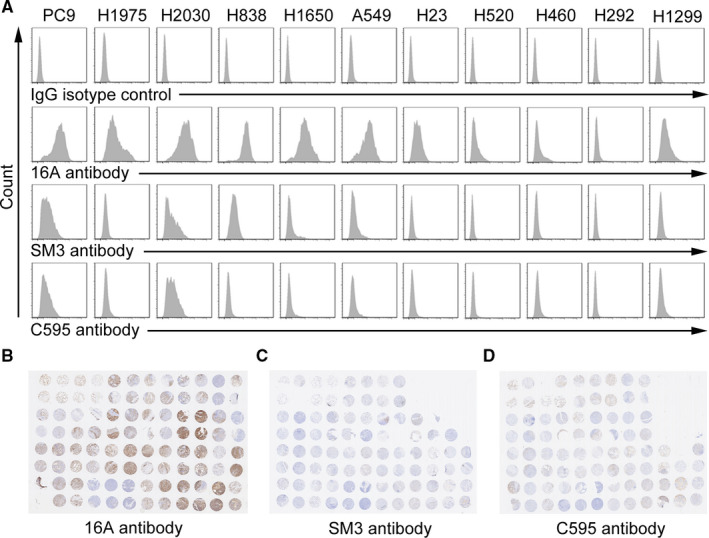
Staining of non‐small cell lung cancer (NSCLC) cells and tissues by 16A (specific to GSTA neoantigen), SM3, and C595 (specific to PDTR neoantigen). (A) NSCLC cell lines were stained with 16A, SM3, and C595 antibodies. (B) Tissue array slides containing consecutive tissue sections from the same NSCLC patients were stained with 16A, SM3, and C595 antibodies.

Flow cytometry analysis revealed that the 16A antibody, which specifically binds to the GSTA neoantigen^21^ displayed high positivity in 11 NSCLC cell lines (Figure 2A). In contrast, SM3 and C595, two antibody clones specifically binding to the PDTR neoantigen epitope, displayed extremely low positivity in NSCLC cell lines. We further confirmed this finding through immunohistochemical staining in consecutive sections of tumor tissue specimens harvested from NSCLC patients. Tumors from the same patients displayed a high staining intensity for the 16 antibody but were negative for SM3 or C595 (Figure 2B–D).

**Figure 3:**
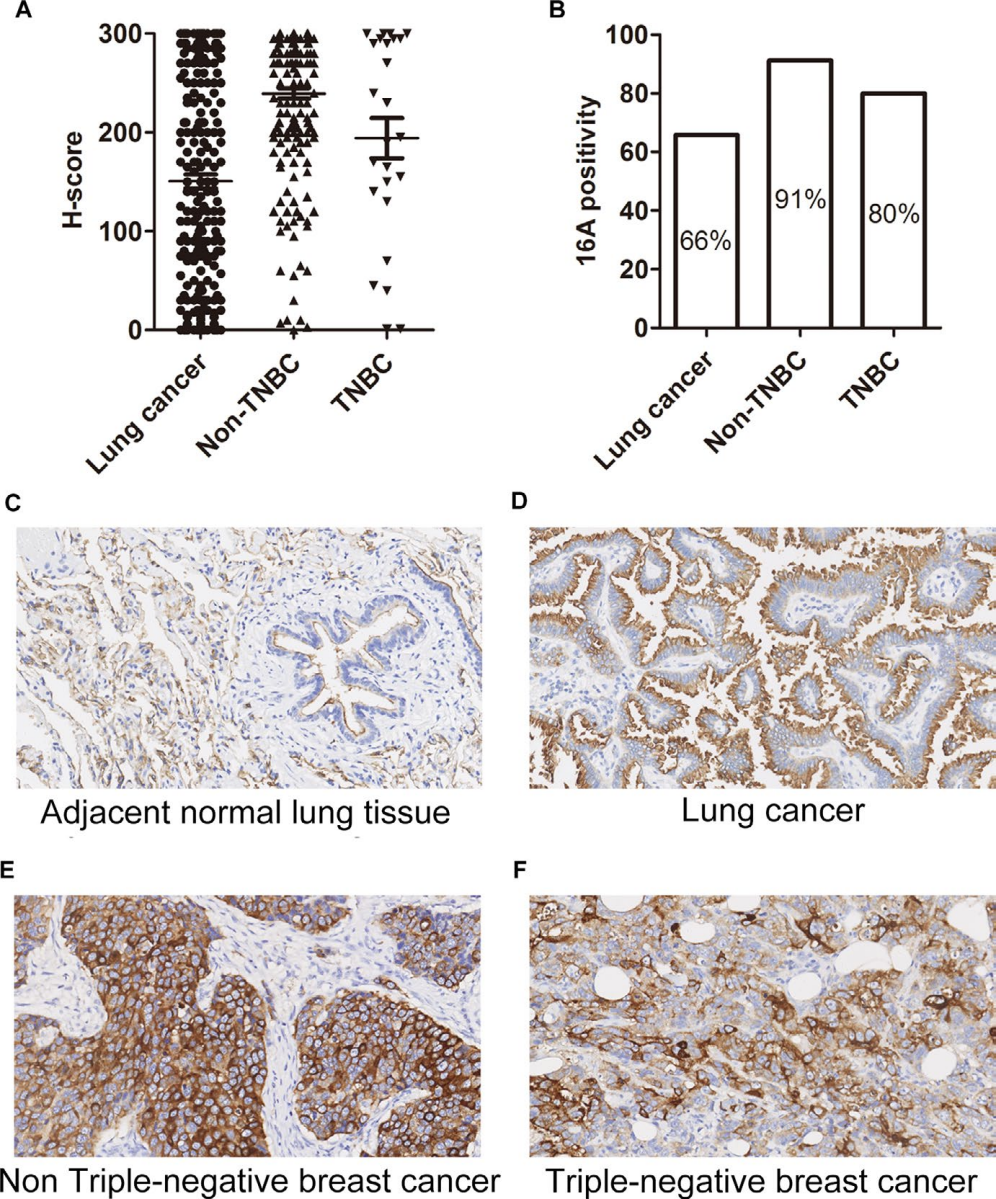
Expression of the aberrantly glycosylated MUC1 peptide motif in lung and breast cancer tissues upon 16A antibody staining. (A) H‐score of the cancer tissue array stained with the 16A antibody. The intensity of IHC staining was scored at four levels: 0 (negative), 1 (weak staining), 2 (medium staining), and 3 (strong staining). The percentages of tumor cells at different intensity levels were evaluated. Total Score = (% at 0) ×0 + (% at 1) × 1 + (% at 2) ×2 + (% at 3) × 3. (B) 16A positivity in lung cancer, breast cancer, and triple‐negative breast cancer (TNBC) samples. (C, D, E, F) Representative photographs of MUC1 immunostaining in peritumoral (C), lung cancer (D), Non‐TNBC (E), and TNBC (F) tissue with the 16A antibody (original magnification ×200). Positivity was defined as ≥30% of tumor with staining ≥2+.

We and others previously reported that antibodies specific to tumor MUC1 preferentially bind to the tumor cell surface, but not healthy cells.^2^ IHC revealed strong binding of 16A mAb to lung (Figure 3A; Figure S1), breast, triple‐negative breast cancer (TNBC; Figure 3A; Figure S2), and gastric (Figure S3) cancer tissues. Positivity of 16A mAb staining was >60% in lung, breast, TNBC (Figure 3B; Table 1), and gastric cancers (Figure S3; Table 1). Weak binding was noted in colon and rectum cancer tissues for the 16A antibody (Figure S3; Table 1). Significantly higher expression was observed in lung adenocarcinoma and squamous carcinoma, relative to peritumoral tissues (Figure S4).

**Table 1:** Positivity of 16A staining in solid tumors

Solid tumors	Ratios (%)
Lung cancer	65.8
Non‐triple‐negative breast cancer	91.2
Triple‐negative breast cancer	80
Breast cancer	90
Stomach cancer	67.1
Colon cancer	28.5
Rectum cancer	19.7

16A staining was observed in the cytoplasm of peritumoral cells (Figure 3C); however, staining was very weak at the cell surface. In contrast, strong staining for 16A was observed on the cell surface of tumor cells (Figure 3D). Moreover, strong 16A staining was observed in both the cytoplasm and cell surface of breast and TNBC cells (Figure 3E,F).

Normal tissues including thymus, tonsil, spleen, cerebellum, pituitary, ovary, liver, paranephros, testis, intestine, cervix, salivary gland, bone marrow, cerebral cortex, bladder, striated muscle, and heart displayed weak or no binding to 16A (Figure S5). Strong cytoplasmic staining was observed in the stomach, ureter, pancreas, and endometrium.

### 3.3 Drug antibody ratio of 16A‐MMAE

**Figure 4:**
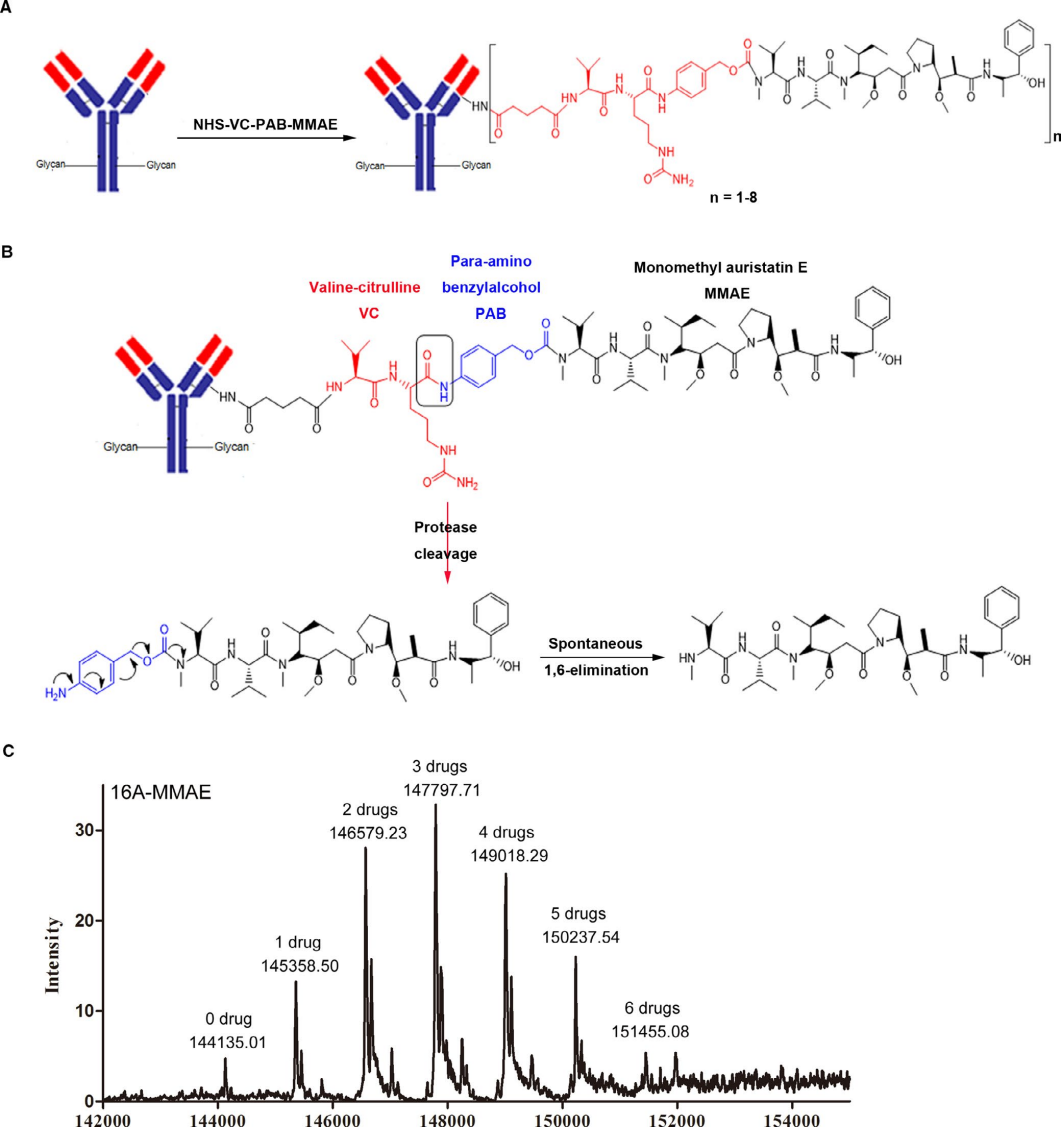
Preparation of the Val‐Cit‐MMAE antibody‐drug conjugate (ADC) and drug antibody ratio of 16A‐MMAE. (A) A schematic representation of the chemical synthesis of the antibody‐Val‐Cit‐MMAE ADC. (B) A schematic representation of Val‐Cit linker cleavage by cathepsin B after ADC internalization by tumor cells. The activated MMAE drug formed from spontaneous 1,6‐elimination. (C) Liquid chromatography–mass spectrometry analysis of 16A‐MMAE. The average drug antibody ratio was 3.11.

MMAE, a synthetic analog of the natural product dolastatin 10, originally isolated from sea hare, is a potent inhibitor of tubulin polymerization. We synthesized 16A‐MMAE using a cleavable Val‐Cit dipeptide linker^25^ connecting a payload with a *p*‐aminobenzyloxycarbonyl (PABC) group (Figure 4A). Val‐Cit linkers are selectively cleaved by lysosomal enzymes upon 16A‐MMAE internalization by cancer cells, resulting in MMAE release (Figure 4B). LC‐MS analysis was performed to determine the average drug antibody ratio (DAR) of 16A‐MMAE. The conjugation mixture contains 16A conjugates with 0, 1, 2, 3, 4, 5, and 6 drugs per antibody, and the average DAR of 16A‐MMAE was 3.11 (Figure 4C). The heterogeneity of DAR values is a common property of lysine‐linked ADCs, including the T‐DM1 reported in previous studies.^26^, ^27^ In our experiment, we maintained consistent conjugation conditions (temperature, solvent, pH, concentration, and the ratio of materials). Furthermore, we combined real‐time DAR monitoring in accordance with our previous study,^28^ to prevent batch‐to‐batch inconsistency.

### 3.4 Pharmacokinetics of 16A‐MMAE

**Figure 5:**
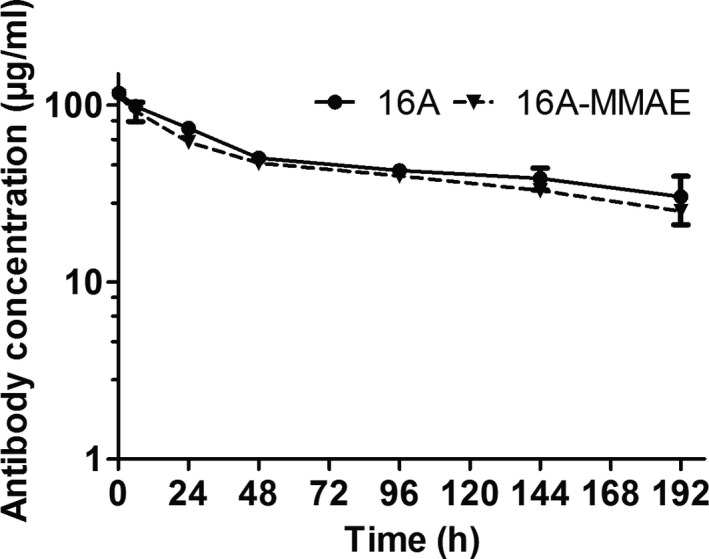
Pharmacokinetic profiles of nonconjugated 16A and 16A‐MMAE in vivo. C57BL/6 mice were injected intravenously with a single dose of nonconjugated 16A mAb or 16A‐MMAE ADC at 5 mg/kg. The serum antibody concentration was determined through ELISA at different time points.

To determine the serum half‐life of nonconjugated 16A antibody and 16A‐MMAE in C57BL/6 mice, we intravenously injected 16A or 16A‐MMAE and measured the serum antibody concentration at the different time points (0.25, 6, 24, 48, 96, 144, and 192 h) (Figure 5). Nonconjugated 16A antibody had a serum half‐life of 207.00 h, and the 16A‐MMAE displayed a shorter serum half‐life (144.22 h, Table S1). The clearance and mean residence durations are consistent with the serum half‐life.

### 3.5 IC_50_ of 16A‐MMAE

The cytotoxicity assay revealed that 16A‐MMAE strongly eliminated tumor cells mostly in the lung, breast, pancreatic, gastric, and ovarian (Figure S6) cell lines. The IC_50_ of 16A‐MMAE is indicated in Table 2. Flow cytometry revealed that most of the lung, breast, pancreatic, and gastric (Figure S6) cancer cells were intensely stained with the 16A antibody.

**Table 2:** IC_50_ values of 16A‐MMAE for cancer cell lines

Cancer type	Cell line	IC_50_ (nM)
Lung cancer	A549	0.20 ± 0.03
	H838	0.53 ± 0.08
	H1650	0.66 ± 0.11
	H2030	1.06 ± 0.17
	PC9	9.80 ± 1.44
	H1975	49.40 ± 20.27
Breast cancer	MCF‐7	1.58 ± 0.22
	SKBR3	3.34 ± 0.41
Ovarian cancer	H8910	0.62 ± 0.04
	SKOV3	32.13 ± 12.75
	ES2	177.2 ± 6.2
	hey	>700
	KGN	>800
Gastric cancer	HGC‐27	13.24 ± 0.65
	N87	24.04 ± 8.32
Pancreatic cancer	CFPAC1	0.53 ± 0.40
	PANC‐1	4.09 ± 1.12

### 3.6 In vivo antitumoral activity of 16A‐MMAE

**Figure 6:**
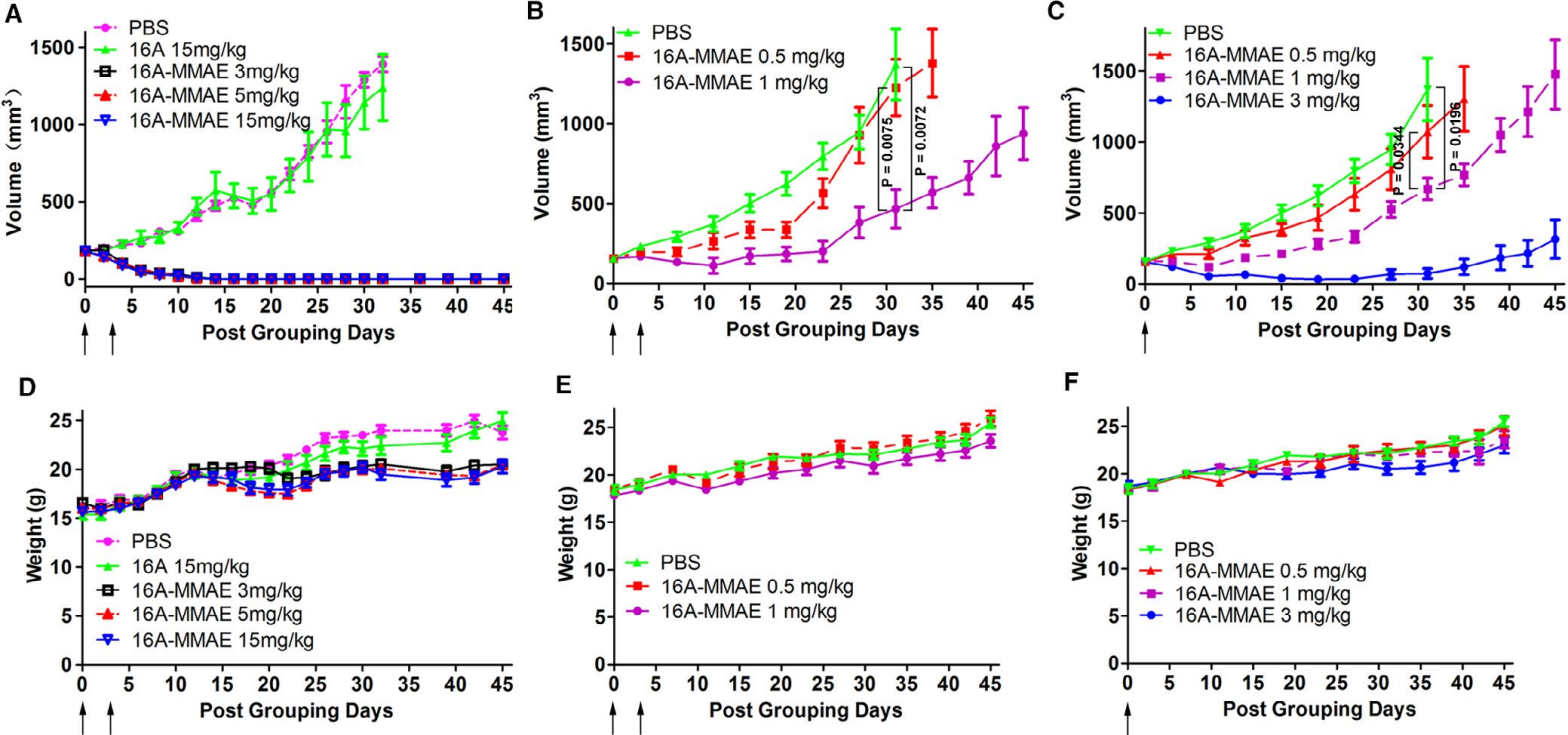
Antitumoral effect of 16A‐MMAE in the H838 mouse xenograft model. (A) Groups of mice (BALB/c nu/nu, female, 6‐week‐old) harboring ~200 mm^3^ H838 tumors were treated with 16A‐MMAE or nonconjugated antibody at 3, 5, or 15 mg/kg, respectively. Arrows indicate time of drug treatment. (B, C) Mice harboring ~200 mm^3^ H838 tumors were treated with 16A‐MMAE (one or two doses) at 0.5, 1, or 3 mg/kg, respectively. Arrows indicate the time of drug treatment. (D, E, F) The body weight of treated mice was measured in all groups. Data points represent mean ± SEM values (*n* = 5)

The in vivo antitumoral activity of 16A‐MMAE was evaluated in aH838 mouse xenograft model. The results show that 16A‐MMAE inhibited tumor growth in mice, in a dose‐dependent manner. Tumors were alleviated after two doses of 3 mg/kg 16A‐MMAE (Figure 6A). The minimal effective dose was 1 mg/kg in two doses (Figure 6B) or a single dose (Figure 6C).

### 3.7 In vivo toxicity of 16A‐MMAE

The in vivo toxicities of the 16A‐MMAE in tumor‐bearing mice were monitored through their body weight. No significant changes were observed in the treated groups upon administration of 5 mg/kg compared to the PBS control (Figure 6D–F). To better assess drug toxicity, hMUC1 transgenic mice^23^ (024631‐C57BL/6‐Tg(MUC1)79.24Gend/J, from Jackson Laboratory) were administered increasing doses of 16A‐MMAE (0, 3, 15, and 30 mg/kg) and euthanized at 3, 14, or 28 days after drug treatment. Tissue sections of the major organs (heart, liver, spleen, lung, kidney, stomach, intestine, and pancreas) were analyzed after H&E staining. No obvious pathological changes were observed at the aforementioned three time points in the group administered a 3 mg/kg dose. For the 15 and 30 mg/kg groups, minor pathological changes were observed in some tissues (liver, lung, and kidney; Figure S7B; Figure S7C). Immunohistochemical staining of normal hMUC1‐Tg mouse tissues with the 16A antibody revealed that the target organs of toxicity are partly associated with antibody binding. Binding of the 16A antibody was observed not only in the liver, lung, and kidney, but also in the stomach, colon, cecum, rectum, salivary gland, trachea, uterus, and testis. No 16A antibody staining was observed in the heart, spleen, duodenum, jejunum, ileum, esophagus, brain, adrenal gland, sternum, vagina, oviduct, ovary, skin, bladder, bicipital muscle, epididymis, prostate, and seminal vesicles (Figure S8).

Furthermore, the in vivo antitumoral activity of 16A‐MMAE was assessed using a syngeneic tumor model in hMUC1‐Tg mice using a B16‐OVA cell line stably transfected with human *MUC1*. The results indicate that 16A‐MMAE inhibits tumor growth in mice in a dose‐dependent manner (Figure S9). Tumors could be inhibited with two doses of 16A‐MMAE at 10 mg/kg.

## 4 DISCUSSION

Tissue positivity is critical for the development of antibody‐based therapeutics. The potential for posttranslational modification of glycoproteins has received increasing attention in cancer therapy. For example, the most common glycan structures in lung cancer include the Tn antigen (GalNAc), STn antigen (Neu5Acα2‐6GalNAc), and ST antigen (NeuAcα1‐3Gaβ1‐3GalNAc).^29^ However, the exact glycopeptide sequences in every individual cancer patients are highly variable and diverse, owing to the “assembly line” nature of glycosylation pathway. Big data on the glycopeptide epitopes caused by such aberrant glycosylation on specific glycoproteins in cancer populations are unavailable. We previously predicted the glycopeptide sequences in lung cancer by MATLAB software.^21^ However, these sequences have not been verified through mass spectrometry. The availability of monoclonal antibodies specific to synthetic glycopeptides partially addresses this issue as immunohistochemical staining by such monoclonal antibodies supports the expression of a glycopeptide sequence, despite potential cross‐reactivity of the respective monoclonal antibody to other structurally related glycopeptide sequences.

This study shows that 16A mAb, which targets the GSTA motif of MUC1,^21^ broadly binds to various cancer cells, including TNBC and gastric cancer. The positivity of 16A mAb staining in breast cancer (90%) is greater than that of SAR566658,^30^, ^31^, ^32^ which binds to a sialylated unknown MUC1 sequence in bladder, breast, ovary, pancreatic, head, and neck cancers with positivity rates of 59%, 29%–35%, 70%, 59%, and 17%, respectively. For cancer tissues negative on 16A mAb staining, other mAbs targeting PDTR or GVTS motifs are worth further investigation.

Several ADCs reportedly target MUC1. Lovat et al. reported the efficacy of the hHuHFMG1 ADC in esophageal adenocarcinoma.^33^ Kufe et al. reported an ADC targeting the non‐glycosylated C‐terminal of MUC1.^34^ Antitumor efficacy and toxicity are two major criteria to be assessed for their clinical application. A phase I clinical trial on SAR566658 administered among patients with CA6‐positive ovarian, pancreatic, and breast tumors has been completed.^30^, ^31^, ^32^ SAR566658 is CA6 mAb conjugated to DM4, a maytansinoid derivative, by SPDB (N‐succinimidyl‐4‐(2‐pyridyldithio) butanoate) linker, a hindered disulfide bond stable linker which is stable in the blood. A partial response was obtained for breast, ovarian, and lung cancers. Dose‐limiting toxicities were observed at 240 mg/m^2^ (diarrhea and keratitis). Late occurrence of reversible corneal toxicity was observed at >150 mg/m^2^.

In this study, we used a hMUC1 transgenic mouse model to further assess the toxicity of 16A‐MMAE. Our safety data show that 16A‐MMAE was well‐tolerated in hMUC1 transgenic mice at 3 mg/kg. At higher dose, 16A‐MMAE exhibited a dose‐limiting toxicity. However, tissue toxicity is not clearly associated with 16A mAb positivity. However, the mechanism underlying these toxic effects remains unclear. This may occur owing to baseline expression of 16A epitopes in target organs. Alternatively, the toxicity might have resulted from nonspecific MMAE release in the serum, since we used Val‐Cit linkers that can be hydrolyzed in mouse plasma by carboxylesterase 1c.^25^, ^35^

Drug pay load is another critical factor for the toxicity of ADCs. Recently, a topoisomerase I inhibitor payload was successful in clinical trials, including trastuzumab deruxtecan (Her2/Exatecan Antibody‐Drug Conjugate),^36^ Labetuzumab Govitecan (CEACAM5/SN‐38 Antibody‐Drug Conjugate),^37^ and Sacituzumab Govitecan (Trop‐2/SN‐38 Antibody‐Drug Conjugate).^38^ Such moderately cytotoxic pay loads often led to a no‐observed‐adverse‐effect level (NOAEL) in dose escalating studies, thus, serving as promising conjugates for mAbs specific to cancer glycopeptides. Novel conjugation methods more specific and potent in conjugating drug payloads to antibodies have been evolving.^39^ More interestingly, non‐internalizing ADCs releasing their drug components through a click reaction with a chemical activator have been recently developed, which are effective in treating cancers resistant to those ADCs.^40^

In summary, this study reports a neoantigen epitope generated through aberrant posttranslational modification of glycoproteins, with high positivity in numerous cancer types. 16A antibody preferentially binds to the cell surface of cancer cells, although its binding with normal cells cannot be absolutely excluded. A balance between antitumoral efficacy and toxicity can further be fine‐tuned by optimizing the linker and the drug payload.

## CONFLICT OF INTEREST

The authors declare no conflict of interest.

## AUTHOR CONTRIBUTIONS

Dapeng Zhou, Patrick Hwu, and Wei Huang designed this study. Deng Pan, Yubo Tang, Jiao Tong, Chengmei Xie, Jiaxi Chen, Chunchao Feng, Patrick Hwu, and Wei Huang contributed to the collection, analysis, and interpretation of data. Deng Pan and Dapeng Zhou wrote the manuscript. All authors read and approved the final manuscript.

## Supporting information

Supplementary Material

## Data Availability

All data generated or analyzed during this study are included in this published article (and its supplementary information files).
