# Supplementary material for: An antibody‐drug conjugate targeting a GSTA glycosite‐signature epitope of MUC1 expressed by non‐small cell lung cancer
Source: Cancer Med. 2020 Oct 20;9(24):9529–40. doi: 10.1002/cam4.3554 (PMC7774737; doi:10.1002/cam4.3554)
Supplement: Supplementary file 1 — Supplementary Material [file CAM4-9-9529-s001.docx]

**Supplemental Online Figure Legends:**

**Figure S1: Immunohistochemical staining of tissues from lung cancer patients with the 16A antibody.**

Lung cancer tissue slides were obtained from Pantomics (Richmond, CA). Slides were stained by Crownbio, China.

**Figure S2: Immunohistochemical staining of tissues from breast cancer patients with the 16A antibody.**

Breast cancer tissue slides were obtained from Pantomics (Richmond, CA, USA). Slides were stained by Crownbio, China.

**Figure S3: Immunohistochemical staining of gastric, colon, and rectum cancers with the 16A antibody.**

Cancer tissue slides were obtained from Pantomics (Richmond, CA, USA). Slides were stained by Crownbio, China.

**Figure S4: Immunohistochemical staining of paired lung cancer and peritumoral tissues with the 16A antibody.**

Slides containing paired lung cancer and peritumoral tissues were obtained from Pantomics (Richmond, CA, USA). Slides were stained by Crownbio, China.

**A),B)**Human tissue array containing multiple tissues from lung adenocarcinoma and squamous carcinoma patients were stained with the 16A antibody. a1 and a2 (b1 and b2) are tumor tissue, a3 (b3) is the same patient’s peritumoral tissue.

**Figure S5: Immunohistochemical staining of tissues from healthy individuals with the 16A antibody.**

Slides containing multiple types of tissues from healthy individuals were obtained from Pantomics (Richmond, CA, USA). Slides were stained by Crownbio, China.

**Figure S6: *In vitro* antitumoral efficacy of 16A-MMAE.**

Up panels: inhibition of cancer cell lines by increasing the concentrations of 16A-MMAE.

Lower panels: flow cytometry staining of cell lines with the 16A antibody.

**Figure S7: Toxicity of 16A-MMAE in hMUC1 transgenic mice.**

16A-MMAE was administered to hMUC1 transgenic mice (n = 6 per group, three male and three female) via the tail vein at a single dose of 0, 3, 15, or 30 mg/kg. Tissues were harvested for clinicopathological assessment on days 3, 14, and 28 (two mice per group at each time point, one male and one female). Histopathological changes in the heart, liver, spleen, lung, kidney, gastric, pancreatic, and small intestine were examined after H&E staining (original magnification, ×100).

**Figure S8: Immunohistochemical staining of multiple organs of the hMUC1 transgenic mice and wild type control with the 16A antibody.** Transgenic (hMUC1) and wild type mouse tissues (heart, liver, spleen, lung, kidney, pancreas, stomach, duodenum, jejunum, ileum, colon, cecum, rectum, esophagus, brain, salivary gland, trachea, adrenal gland, sternum, vagina, oviduct, ovary, uterus, skin, eyeball, bladder, bicipital muscle, epididymis, testis, prostate, and seminal vesicle) were stained with the 16A antibody (original magnification, ×100).

**Figure S9. Antitumoral effect of 16A-MMAE in B16-OVA-hMUC1 model.** The *in vivo* antitumoral activity of 16A-MMAE was evaluated in a B16-OVA-hMUC1 cell transplant model *in vivo* using C57BL/6-Tg(MUC1)79.24Gend/J mice. 16A-MMAE inhibited tumor growth in mice in a dose-dependent manner. The tumor could be inhibited with two doses of 16A-MMAE at 10 mg/kg.

Figure S1


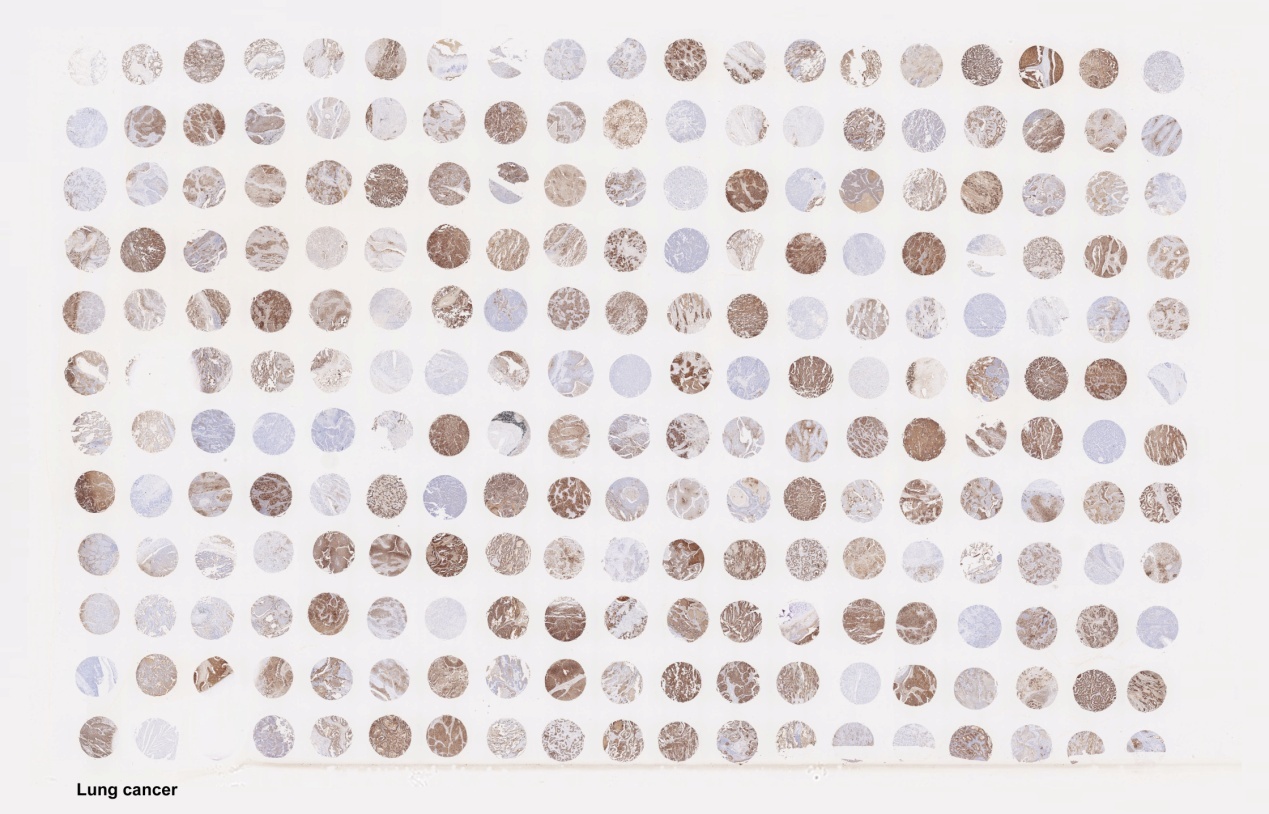


Figure S2


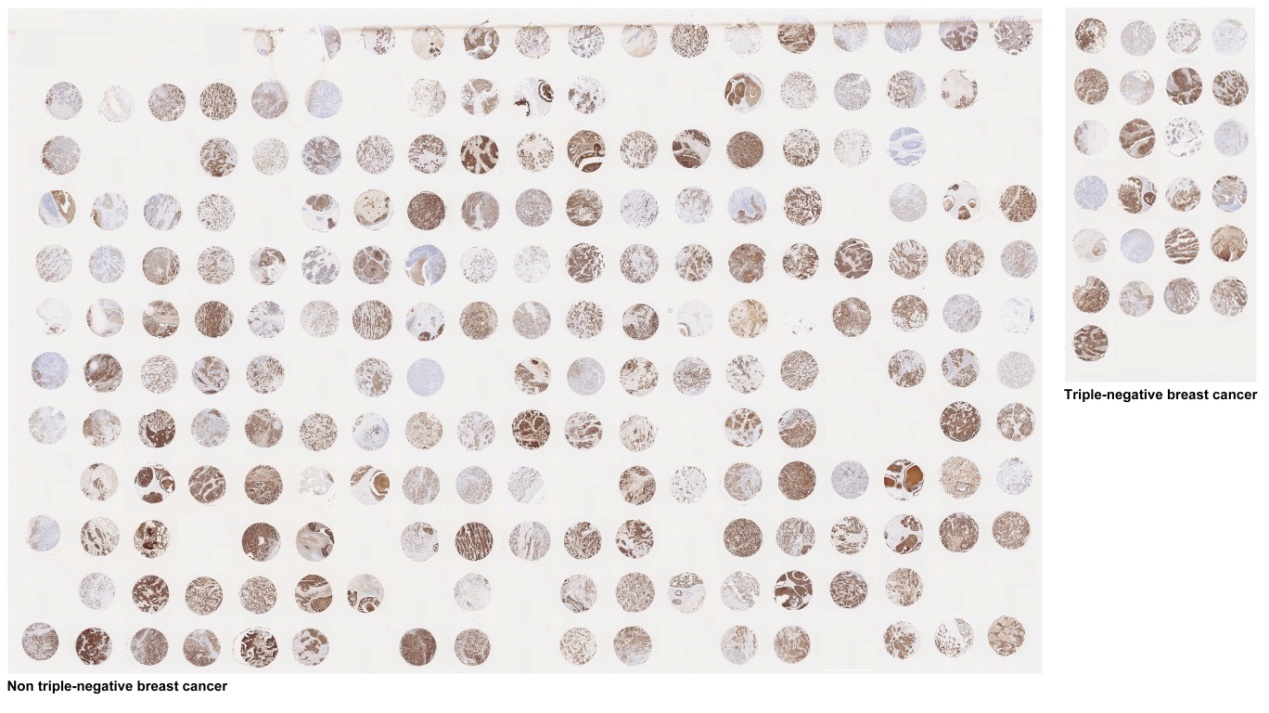


Figure S3


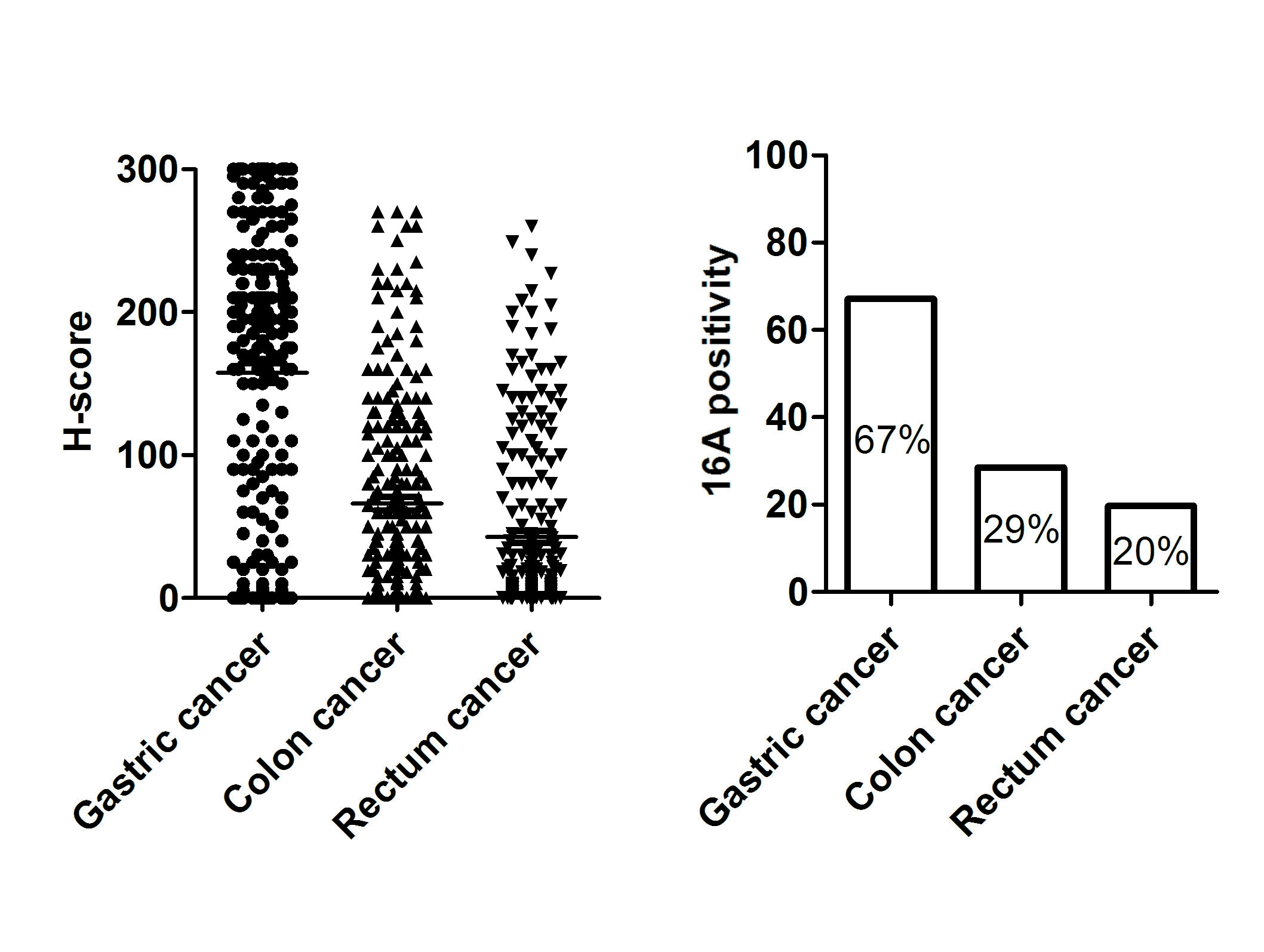


Figure S4


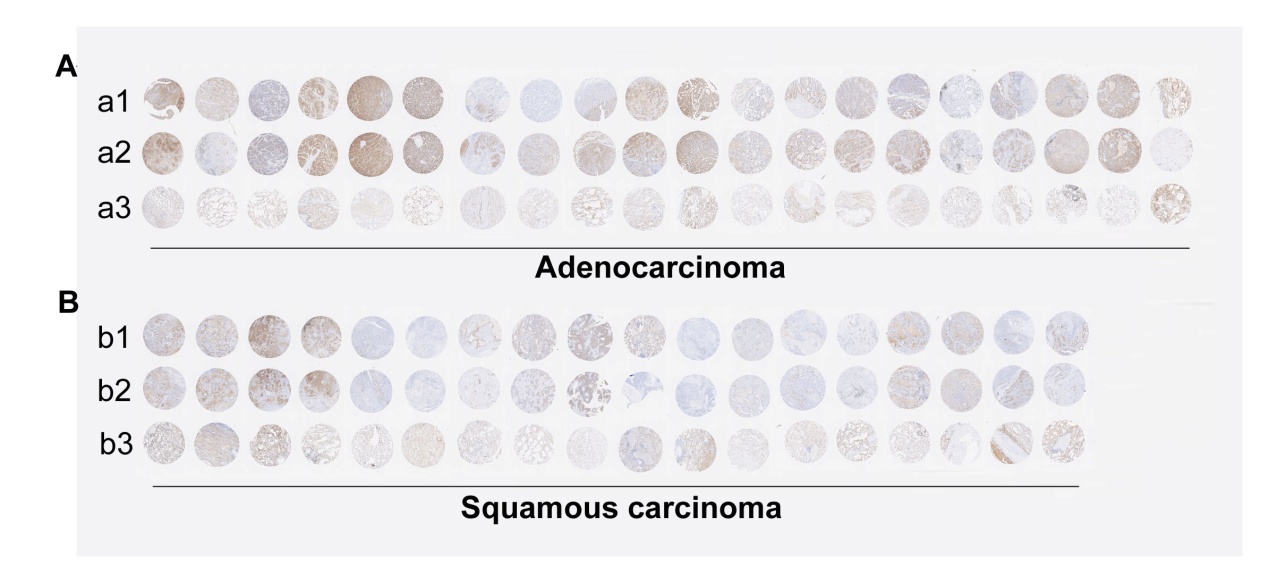


Figure S5


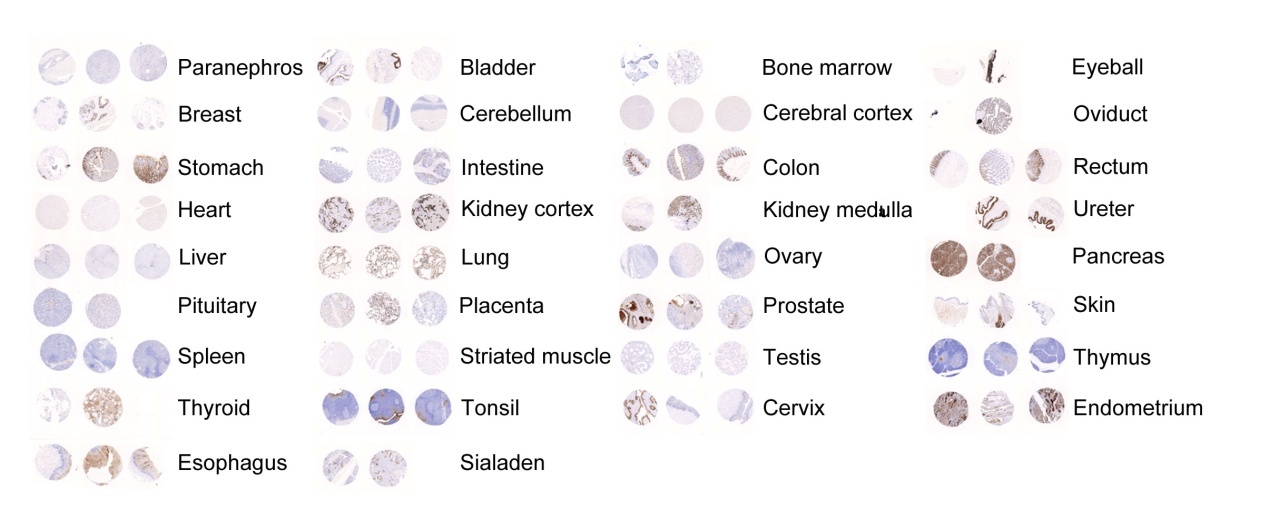


Figure S6


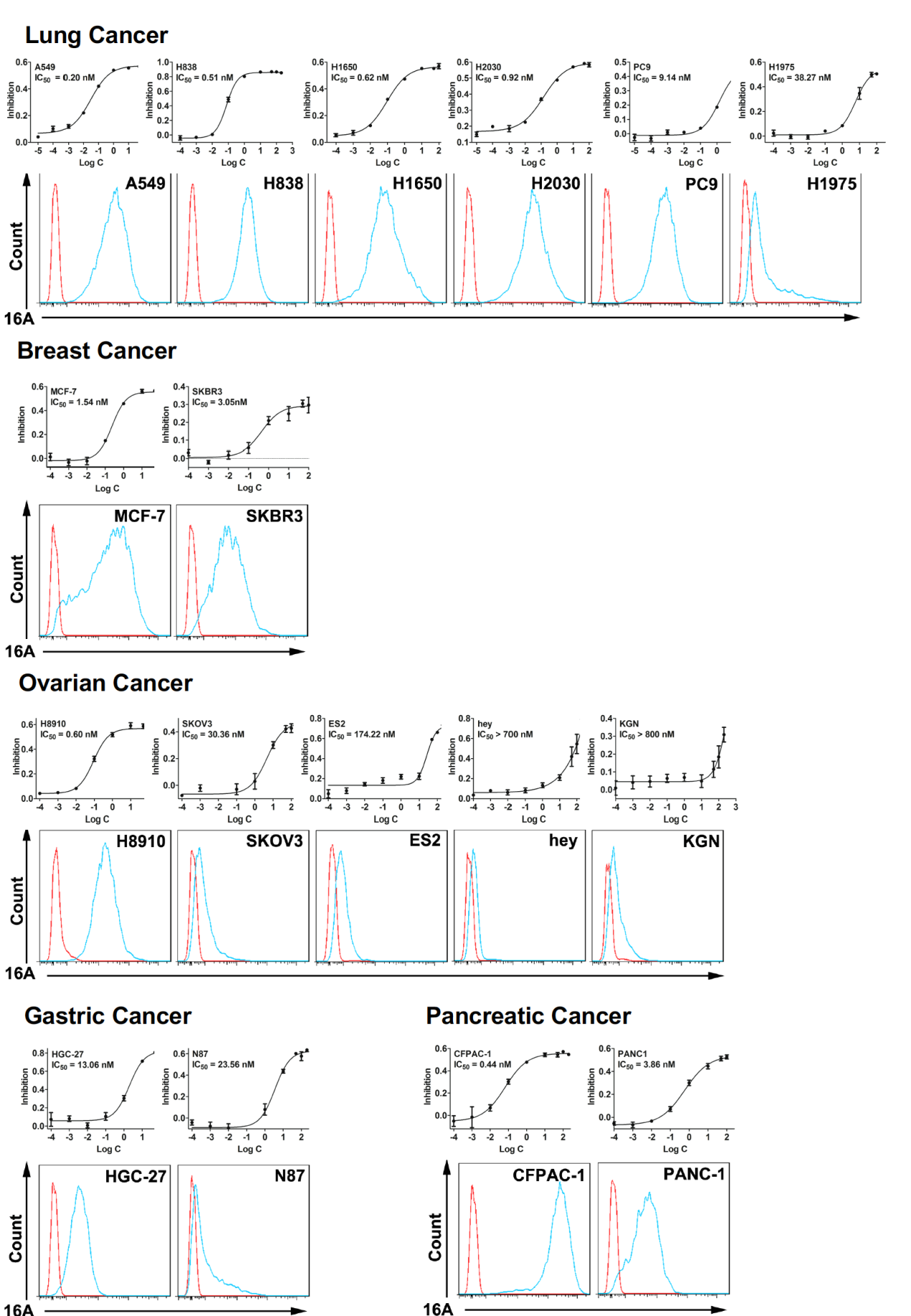


Figure S7A


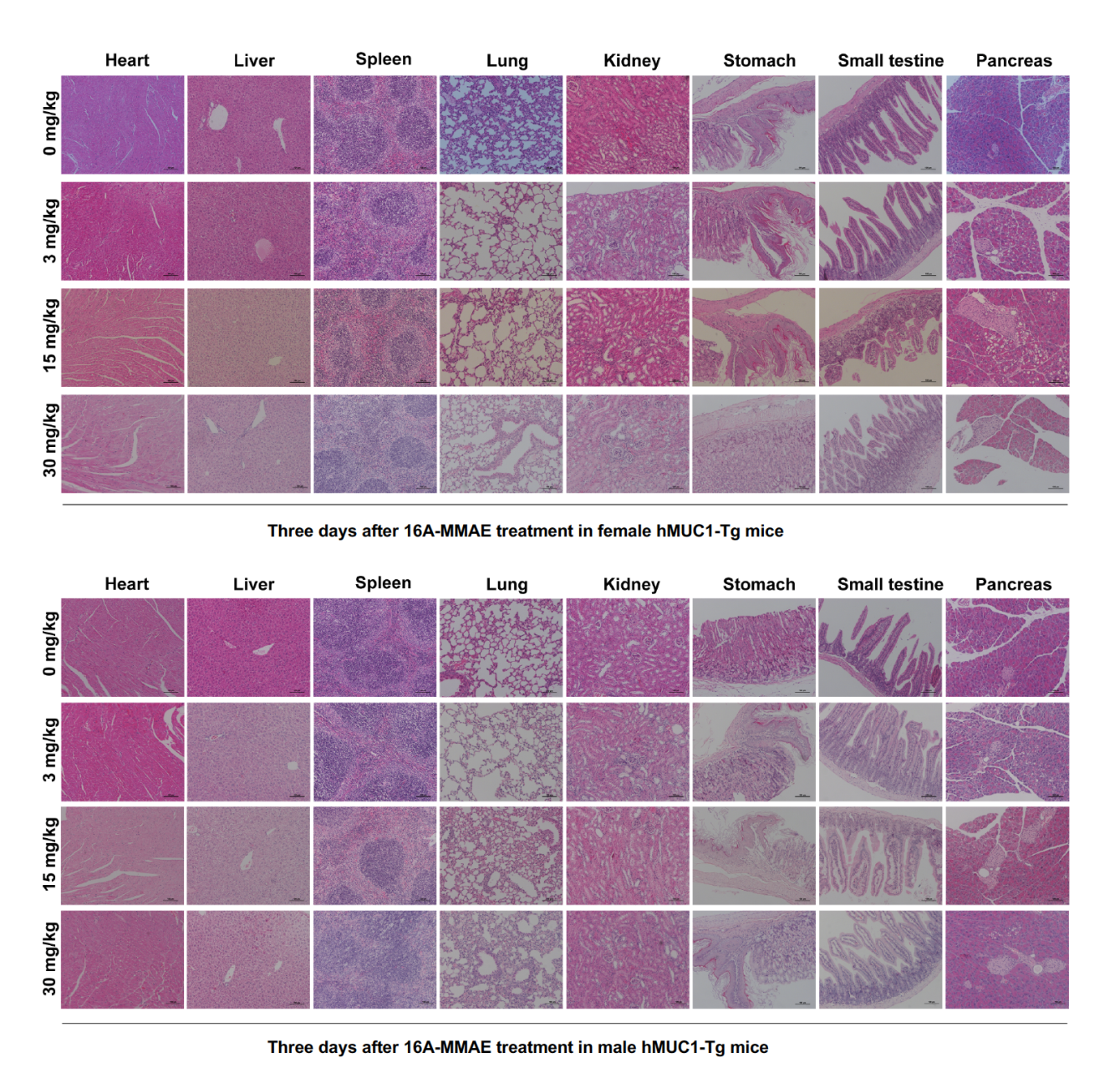


Figure S7B


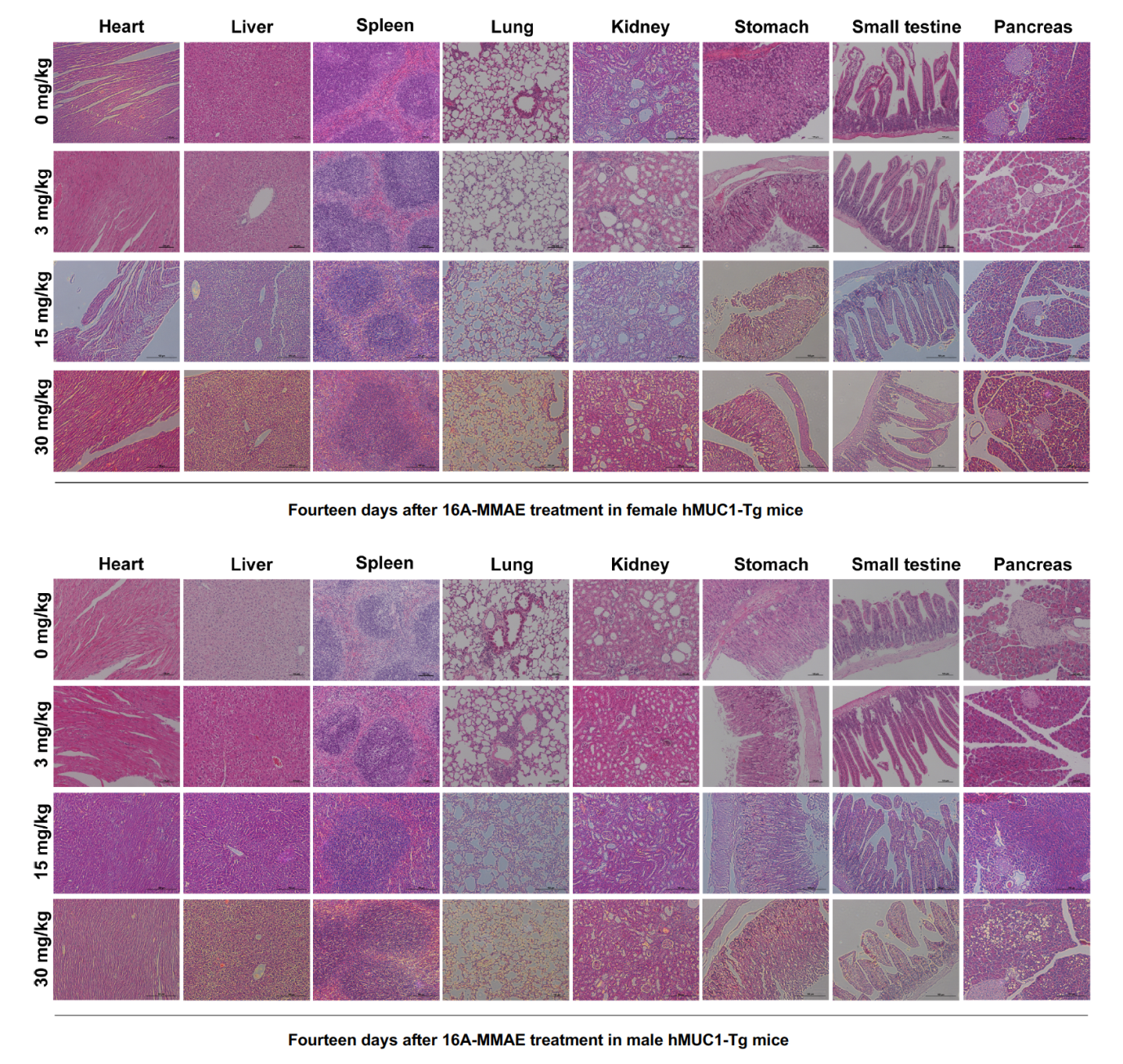


Figure S7C


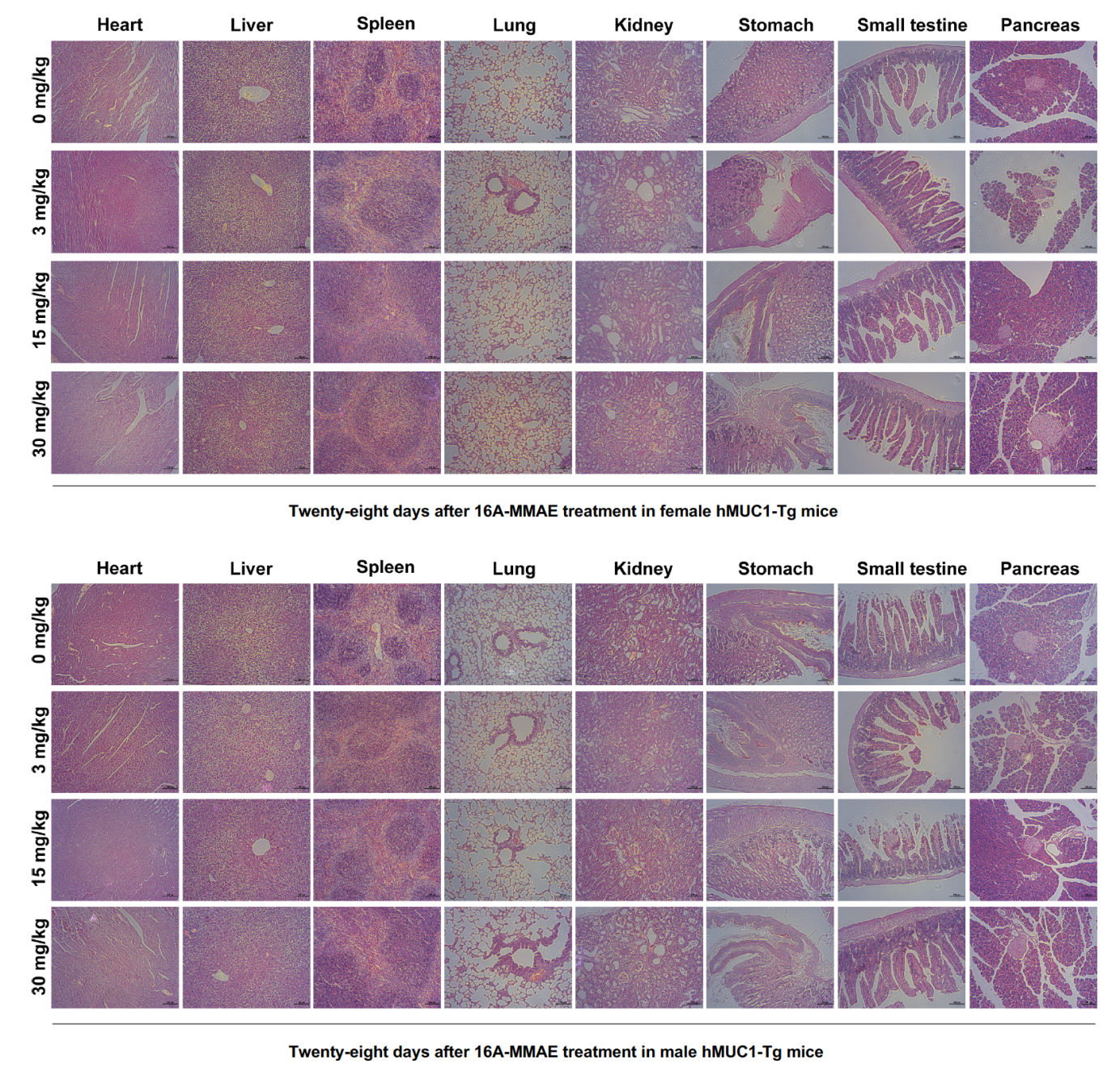


Figure S8


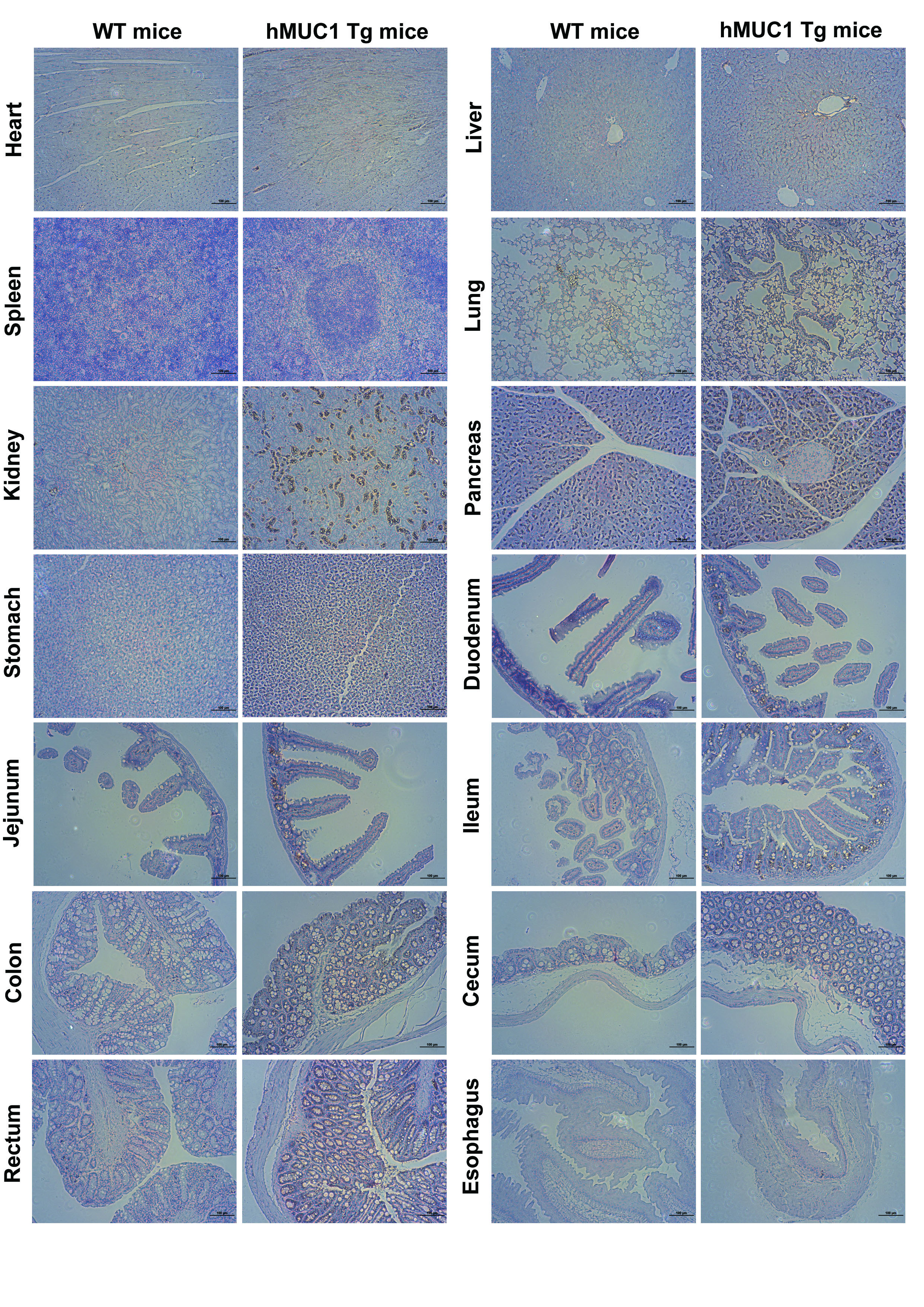


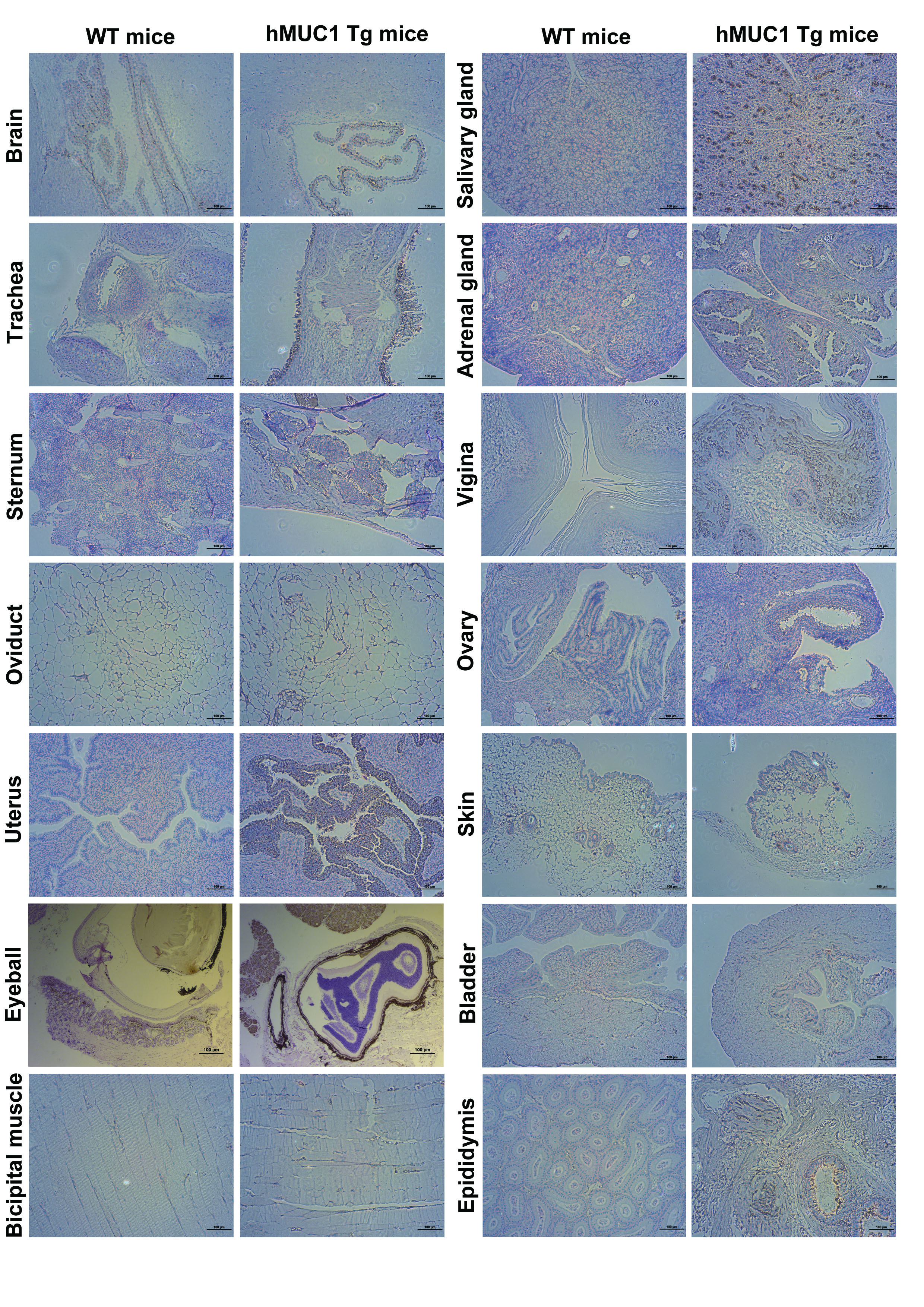


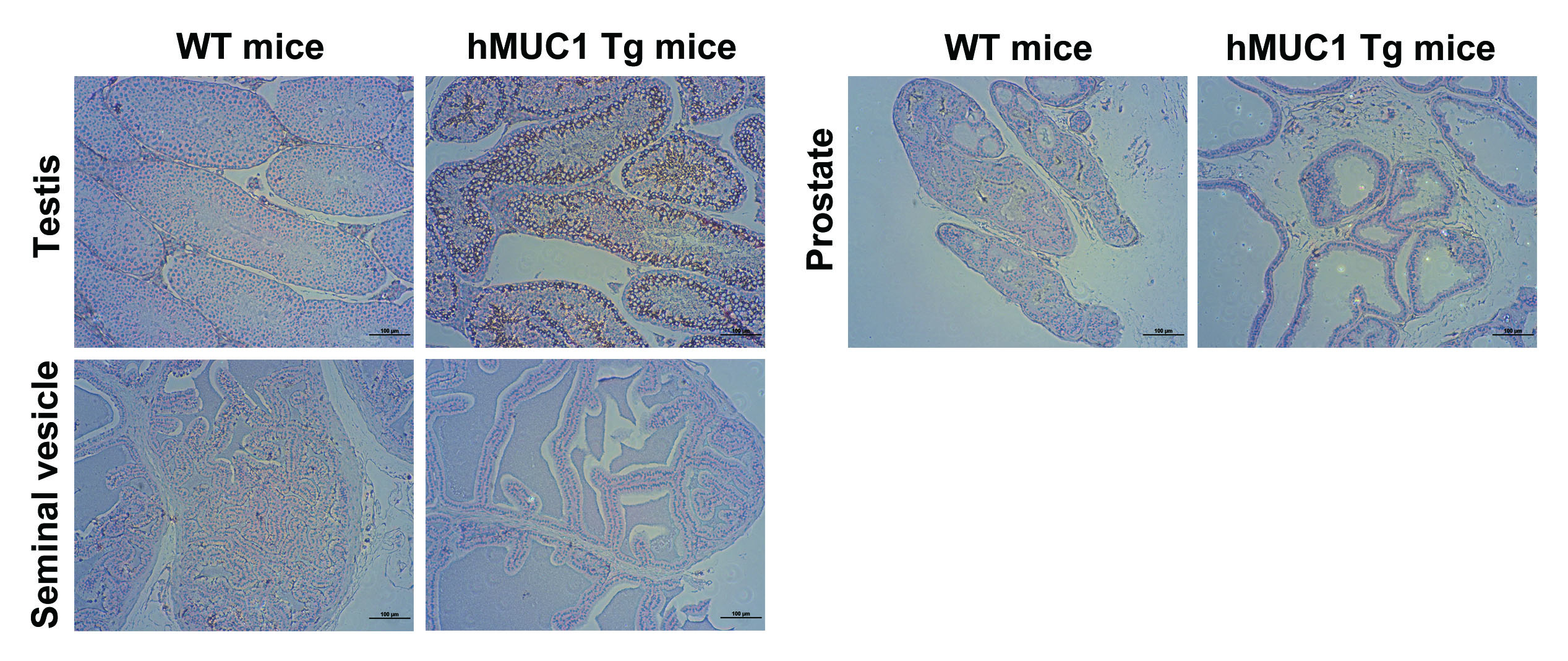


Figure S9


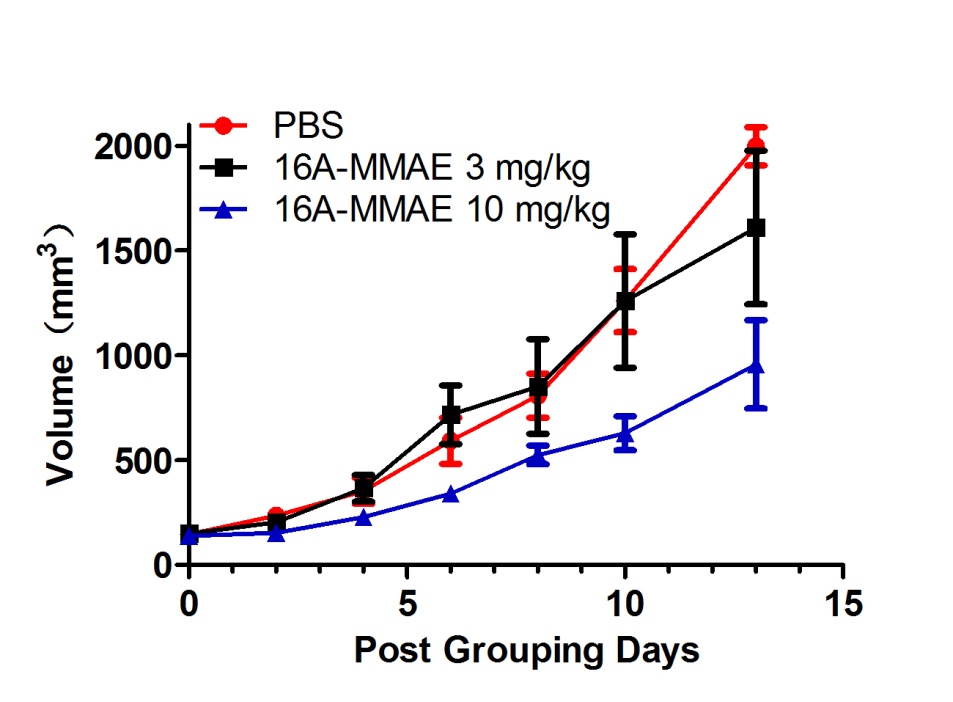


**Table S1. Pharmacokinetics of the 16A-MMAE antibody-drug conjugate.**

| Parameters | 16A | 16A-MMAE |
| --- | --- | --- |
| t_1/2_ (h) | 207.00 | 144.22 |
| CL (ml/h/kg) | 0.27 | 0.36 |
| MRT (h) | 278.87 | 198.98 |

t_1/2_: Elimination Half-life.

CL: Clearance.

MRT: Mean Residence time.
